# Attention-Deficit/Hyperactivity Disorder Symptoms and Later E-Cigarette and Tobacco Use in US Youths

**DOI:** 10.1001/jamanetworkopen.2024.58834

**Published:** 2025-02-11

**Authors:** Sean Esteban McCabe, Emily Pasman, Timothy Wilens, Carol J. Boyd, Philip Veliz, Vita McCabe, Bingxin Chen, Kara Dickinson, Rebecca J. Evans-Polce

**Affiliations:** 1 Center for the Study of Drugs, Alcohol, Smoking and Health, School of Nursing, University of Michigan, Ann Arbor; 2 Survey Research Center, Institute for Social Research, University of Michigan, Ann Arbor; 3 Institute for Research on Women and Gender, University of Michigan, Ann Arbor; 4 Department of Health Behavior and Clinical Sciences, School of Nursing, University of Michigan, Ann Arbor; 5 Department of Psychiatry, Massachusetts General Hospital, Boston; 6 Harvard Medical School, Harvard University, Boston, Massachusetts; 7 Department of Psychiatry, University of Michigan, Ann Arbor; 8 Department of Systems, Populations and Leadership, University of Michigan, Ann Arbor; 9 Applied Biostatistical Laboratory, School of Nursing, University of Michigan, Ann Arbor

## Abstract

**Importance:**

Information about longitudinal associations of attention-deficit/hyperactivity disorder (ADHD) diagnosis, symptom severity, pharmacotherapy, and incident nicotine and tobacco use, including e-cigarettes, among US youths is limited.

**Objective:**

To evaluate whether ADHD diagnosis, symptom severity, and pharmacotherapy are associated with incident e-cigarette and tobacco use over a 9-year period.

**Design, Setting, and Participants:**

This longitudinal cohort study followed up a nationally representative sample of US youths and their parents in the Population Assessment of Tobacco and Study via questionnaires from wave 1 (September 2013 to December 2014) to waves 2 to 7 (October 2014 to April 2023). Participants were representative of the US civilian noninstitutionalized population. Youths aged 12 to 17 years at wave 1 (N = 13 572) and their parents were interviewed.

**Exposures:**

Time in years and history of ADHD diagnosis, symptom severity, and pharmacotherapy at wave 1 were used to construct 9 mutually exclusive subgroups of US youths, including those with ADHD diagnosis and pharmacotherapy, ADHD diagnosis without pharmacotherapy, and population controls without ADHD diagnosis; within each of these 3 subgroups, ADHD symptom severity was categorized as none, 1 to 2, or 3 to 4 symptoms.

**Main Outcomes and Measures:**

Incident e-cigarette use, cigarette smoking, other tobacco use, and dual use (e-cigarette and cigarette and/or other tobacco use) in weighted percentages with 95% CIs.

**Results:**

Of the 13 572 youths included in the analysis, 6967 (51.3%; 95% CI, 51.2%-51.5%) were male. An estimated 1881 participants (14.1%; 95% CI, 13.2%-15.0%) were diagnosed with ADHD. Multivariable logistic regression analyses indicated that the adjusted odds of incident e-cigarette use, cigarette smoking, other tobacco use, and dual use did not significantly differ between those with asymptomatic ADHD (with or without pharmacotherapy) compared with population controls. In contrast, all subgroups who had 3 or more ADHD symptoms (with or without pharmacotherapy) had significantly higher adjusted odds of e-cigarette use (adjusted odds ratio [AOR], 1.60; 95% CI, 1.34-2.04), cigarette smoking (AOR, 1.52; 95% CI, 1.22-1.89), other tobacco use (AOR, 1.61; 95% CI, 1.27-2.02), and dual use (AOR, 1.72; 95% CI, 1.38-2.14) compared with youths with asymptomatic ADHD or population controls. Among youths with ADHD, those with highly symptomatic ADHD were significantly more likely to initiate e-cigarette (AOR, 1.68; 95% CI, 1.16-2.44) and dual use (AOR, 1.82; 95% CI, 1.17-2.83) than youths without symptoms.

**Conclusions and Relevance:**

In this cohort study of US youths, ADHD symptoms were associated with the onset of nicotine and tobacco use. Findings highlight the importance of early diagnosis and effective treatment of ADHD to alleviate symptoms and reduce the risk of later nicotine and tobacco use.

## Key Points

**Question:**

Do youths with asymptomatic attention-deficit/hyperactivity disorder (ADHD) differ from youths with symptomatic ADHD and population controls without ADHD in their risk of incident e-cigarette and tobacco use?

**Findings:**

In this cohort study of 13 572 US youths, odds of e-cigarette use, cigarette smoking, other tobacco use, and dual use did not significantly differ between those with asymptomatic ADHD (with or without pharmacotherapy) and population controls. In contrast, all youths who had 3 or more ADHD symptoms (with or without pharmacotherapy) had significantly higher adjusted odds of using nicotine and tobacco products.

**Meaning:**

These findings highlight the importance of early diagnosis and effective treatment of ADHD to mitigate symptoms and reduce the risk of later nicotine and tobacco use.

## Teaser

This cohort study of data over a 9-year period evaluates whether diagnosis of attention-deficit/hyperactivity disorder, symptom severity, and pharmacotherapy are associated with incident e-cigarette and tobacco use.

## Introduction

Tobacco use remains the leading cause of preventable death in the US and worldwide.^1,2,3^ While associations between attention-deficit/hyperactivity disorder (ADHD) and tobacco use are well-established,^4,5,6,7,8,9,10,11^ the landscape of ADHD, nicotine, and tobacco use among US youths has changed over the past decade.^12,13,14^ For instance, the prevalence of electronic nicotine delivery systems (e-cigarettes) surpassed cigarette smoking among US youths in 2018, and e-cigarette use is now the second most prevalent substance use behavior.^14,15^ The prevalence of ADHD diagnoses and the prescribing of stimulant and nonstimulant pharmacotherapy for ADHD have also increased over the past decade.^12,14^

ADHD is characterized by functional impairment, inattention, and/or hyperactivity and is one of the most common psychiatric disorders among US children and adolescents, with the prevalence increasing over the past 2 decades.^16,17,18^ In 2022, approximately 1 in 9 US youths (11.4%) had received an ADHD diagnosis in their lifetime, and approximately 1 in 10 (10.5%) had a current ADHD diagnosis.^13^ Among US youths with current ADHD, 53.6% used ADHD pharmacotherapy and 44.4% received psychosocial treatment to manage their ADHD symptoms.^10^

A large body of research has established an association between ADHD and tobacco use.^4,5,6,7,8,9,10,11^ For example, a meta-analytic review^4^ concluded that youths with ADHD had more than double the odds of nicotine use by middle adolescence relative to their peers without ADHD. Moreover, a population-based prospective Swedish twin study^5^ followed up nearly 1500 pairs of twins from childhood to adolescence and found that hyperactive and/or impulsive symptoms of ADHD were associated with early onset-tobacco use. Early onset of tobacco use can serve as an important antecedent to substance use disorder in US youths with ADHD and can offer a useful signal to intervene before substance use disorder develops.^19,20,21^

Some experts have asserted that alleviating ADHD symptoms may reduce impulsivity and engagement in substance use and other risky behaviors.^22,23^ However, there are no known national prospective probability-based US studies examining the association between childhood ADHD and later nicotine or tobacco use that have parsed out the nuances of ADHD treatment and ADHD symptom severity. Within this context, prospective research examining the longitudinal associations among ADHD diagnosis, pharmacotherapy, inattentive symptoms, hyperactive and/or impulsive symptoms, and later nicotine e-cigarette and tobacco use among US youths is warranted.

This cohort study aims to extend the literature regarding associations between ADHD and tobacco use by using current (to 2023) nationally representative longitudinal data over a 9-year period that accounts for ADHD symptom severity and assesses longitudinal associations with multiple forms of nicotine and tobacco use, including more contemporary smokeless and electronic products. The following research questions were examined in this study: (1) Do youths with asymptomatic ADHD (with or without pharmacotherapy) differ from youths without ADHD or ADHD symptoms in their risk of incident e-cigarette and tobacco use? (2) Are there differences between youths with ADHD symptoms, with or without an ADHD diagnosis, compared with youths without ADHD or ADHD symptoms in their risk of incident e-cigarette and tobacco use? and (3) Do youths with symptomatic ADHD have an increased risk of incident e-cigarette and tobacco use compared with youths with asymptomatic ADHD?

## Methods

In this cohort study, a nationally representative sample of US youths (N = 13 651) aged 12 to 17 years old and their parents (N = 13 588) from the Population Assessment of Tobacco and Health (PATH) Study were surveyed via questionnaires at wave 1 (September 2013 to December 2014) and followed up over 6 additional waves in subsequent years (October 2014 to April 2023). Beginning in wave 2, youths who turned 18 years of age continued to be followed up via the PATH adult survey. At wave 1, household screenings were conducted with computer-assisted personal interviewing to determine eligibility. All surveys were conducted using audio computer-assisted self-interviews in English or Spanish.^24^ The weighted response rate at wave 1 for youths was 78.4%. Weighted response rates at follow-up ranged from 87.3% (wave 2) to 54.3% (wave 7). Additional details regarding interviewing procedures, sampling, and weighting are available elsewhere.^25^ The present study excluded 2.3% of the sample due to missingness on variables at wave 1, resulting in a final analytic sample of 13 572 youths. The study was approved by the Westat Institutional Review Board, and this secondary data analysis study with restricted data was determined exempt from informed consent by the University of Michigan Institutional Review Board because the data were deidentified. We followed the Strengthening the Reporting of Observational Studies in Epidemiology (STROBE) reporting guideline.

### Measures

#### Key Outcome Variables

At each wave, youths reported on lifetime and past-year use of 9 different nicotine or tobacco products: (1) electronic nicotine products such as e-cigarettes, vape pens, personal vaporizers and mods, e-cigars, e-pipes, e-hookahs, or hookah pens (collectively termed e-cigarettes for this study); (2) cigarettes; (3) cigars; (4) little cigars, cigarillos, or filtered cigars; (5) pipes; (6) hookahs; (7) smokeless tobacco; (8) snus; or (9) dissolvable tobacco. As the primary outcomes, we examined the incidence (new onset of use over the follow-up period 2015-2023 or waves 2-7) of 4 nicotine or tobacco outcomes among youths who reported no lifetime use at wave 1: e-cigarette use, cigarette smoking, other tobacco use (cigars, little cigars, cigarillos, or filtered cigars; pipes, hookahs, smokeless tobacco, snus, or dissolvable tobacco), and dual use (both e-cigarettes and cigarettes or tobacco products). Wave 1 prevalence of these 4 outcomes was also examined.

#### Key Exposure Variables

At wave 1, parents self-reported whether their child had a history of an ADHD diagnosis (“Has [Child’s first name] ever been told by a doctor, nurse, or other health professional that [he or she] has ADHD or ADD?” [yes or no]) and whether their child regularly took medication for ADHD (“In the past 12 months, has [Child’s first name] taken medications regularly for ADHD or ADD?” [yes or no]). From these 2 questions, we created 3 mutually exclusive groups: ADHD diagnosis with pharmacotherapy, ADHD diagnosis without pharmacotherapy, and population controls without an ADHD diagnosis or pharmacotherapy.

Youths completed items from the Global Appraisal of Individual Needs (GAIN) assessment,^24,26,27^ which included a list of ADHD symptoms from validated and reliable screeners recommended in the PhenX Toolkit.^28^ The following 4 items from the GAIN externalizing scale assessed *DSM*-based ADHD inattentive and hyperactive and/or impulsive symptoms:

1. “Had a hard time paying attention at school, work, or home?”
2. “Had a hard time listening to instructions at school, work, or home?”
3. “Felt restless or the need to run around or climb on things?”
4. “Gave answers before the other person finished asking the question?”

For each item, respondents indicated whether they had experienced the symptom multiple times in the past month, 2 to 12 months ago, over 1 year ago, or never. The Cronbach α for the 4 ADHD symptoms was 0.73. Based on recommended cut points, we summed the number of past-year symptoms at wave 1 (range, 0-4) and categorized participants into tertiles consisting of no symptoms, 1 to 2 symptoms, and 3 to 4 symptoms.^26^

#### Covariate Variables

Covariates at wave 1 included age (12-14 vs 15-17 years), sex, US region (Northeast, Midwest, South, or West), internalizing symptoms (feeling very trapped, sad, or depressed; trouble sleeping; feeling nervous, anxious, tense, or scared; and being distressed or upset about the past [scored as 0-4]), other externalizing symptoms (lied or conned to get something; bullied or threatened people; or started a physical fight [scored as 0-3]), lifetime nonmedical use of prescription stimulants (methylphenidate and amphetamine products without a prescription or other than prescribed), Hispanic ethnicity, and race. Participants self-reported their race by answering the question “What is your race? Choose all that apply.” The options included American Indian or Alaska Native, Asian Indian, Black or African American, Chinese, Filipino, Guamanian or Chamorro, Japanese, Korean, Native Hawaiian, Other Asian, Other Pacific Islander, Samoan, Vietnamese, White, do not know, or declined to answer. The PATH team combines many of these categories into Asian and Pacific Islander when they make the data available. Race and other covariates were included because they have been shown to be associated with ADHD diagnosis, symptoms, pharmacotherapy, and e-cigarette or tobacco use.^13,14,15,16,17,29,30,31,32,33^

### Statistical Analysis

We first calculated weighted descriptive statistics for the overall sample and by ADHD diagnosis and use of pharmacotherapy at wave 1. We then estimated the prevalence of each of the 4 nicotine or tobacco use outcomes at wave 1 (2013-2014) and the incidence at follow-up waves 2 through 7 (2015-2023). For each incidence outcome, analyses were restricted to the subpopulation of individuals who were at risk for new use at wave 1. For example, analyses examining incidence of e-cigarette use included only those who had not yet used e-cigarettes by wave 1 and analyses examining incidence of cigarette smoking included only those who had not yet smoked cigarettes by wave 1. A sensitivity analysis was conducted by excluding youths who reported any nicotine or tobacco use at wave 1. Finally, discrete time hazard models were fitted to the PATH data to examine the associations of ADHD diagnosis, pharmacotherapy, and symptoms at wave 1 with subsequent incidence of e-cigarette use, cigarette smoking, other tobacco use, and dual use. To do this we created a long-format data file with a row for each person-wave of data contributed. By using a person-wave data format, we were able to use standard multivariable logistic regression models to estimate the discrete time hazard function.^34,35^ All analyses accounted for the complex sample design of the PATH and used the Stata suite of svy commands, version 18 (StataCorp LLC) and balanced repeated replication weights with a 0.3 Fay adjustment. Two-sided *P* < .05 indicated statistical significance.

## Results

The sample of 13 572 US youths with weighted percentages (6967 male [51.3%; 95% CI, 51.2%-51.5%] and 6605 [48.7%; 95% CI, 48.5%-48.8%] female) comprised 6972 (50.4%; 95% CI, 50.3%-50.6%) aged 12 to 14 years and 6600 (49.6%; 95% CI, 49.4%-49.7%) aged 15 to 17 years old. In terms of race and ethnicity, 1665 (9.3%; 95% CI, 8.9%-9.7%) were American Indian or Alaska Native, Native Hawaiian or Other Pacific Islander, or multiracial; 412 (4.7% 95% CI, 4.6%-4.9%), Asian; 2073 (15.3% 95% CI, 15.0%-15.5%), Black or African American; 3897 (22.2% 95% CI, 22.0%-22.3%), Hispanic; and 9422 (70.8% 95% CI, 70.2%-71.4%), White. A total of 1881 US youths (14.1%; 95% CI, 13.2%-15.0%) were diagnosed with ADHD, with 1074 (57.9%; 95% CI, 55.2%-60.5%) having received pharmacotherapy for ADHD at wave 1 (Table 1). The distribution of ADHD inattentive and hyperactive and/or impulsive symptoms was as follows (percentages are weighted): 3527 youths (25.3%; 95% CI, 24.3%-26.3%) reported no ADHD symptoms, 5186 (38.4%; 95% CI, 37.5%-39.3%) reported 1 to 2 ADHD symptoms, and 4840 (36.3%; 95% CI, 35.3%-37.4%) reported 3 or more ADHD symptoms.

**Table 1.** Sample Characteristics of 13 572 US Youths Aged 12 to 17 Years at Wave 1^a^

Wave 1 characteristics	Diagnosis group, unweighted No. (weighted %) [95% CI]				*P* value^b^
	Overall	ADHD with pharmacotherapy	ADHD without pharmacotherapy	No ADHD (population controls)	
ADHD diagnosis					
Yes	1881 (14.1) [13.2-15.0]	1074 (100)	807 (100)	0	<.001
No	11 691 (85.9) [85.0-86.8]	0	0	11 691 (100)	
ADHD pharmacotherapy					
Yes	1074 (57.9) [55.2-60.5]	1074 (100)	0	0	<.001
No	807 (42.1) [39.5-44.8]	0	807 (100)	11 691 (100)	
ADHD symptoms					
3-4	4840 (36.3) [35.3-37.4]	519 (48.9) [45.5-52.3]	351 (44.4) [40.6-48.4]	3970 (34.4) [33.3-35.6]	<.001
1-2	5186 (38.4) [37.5-39.3]	334 (31.7) [29.0-34.6]	270 (33.5) [30.3-36.8]	4582 (39.3) [38.3-40.2]	
None	3527 (25.3) [24.3-26.3]	220 (19.4) [16.9-22.1]	184 (22.1) [19.3-25.2]	3123 (26.3) [25.2-27.5]	
Sex					
Female	6605 (48.7) [48.5-48.8]	320 (30.1) [27.1-33.2]	259 (32.2) [29.1-35.4]	6026 (51.5) [51.0-51.9]	<.001
Male	6967 (51.3) [51.2-51.5]	754 (69.9) [66.8-72.9]	548 (67.8) [64.6-70.9]	5665 (48.5) [48.1-49.0]	
Race					
American Indian or Alaska Native, Native Hawaiian or Other Pacific Islander, or multiracial	1665 (9.3) [8.9-9.7]	129 (8.6) [7.1-10.4]	116 (11.0) [9.0-13.4]	1420 (9.2) [8.7-9.8]	<.001
Asian	412 (4.7) [4.6-4.9]	16 (2.6) [1.3-3.6]	12 (1.9) [1.2-3.2]	384 (5.2) [5.0-5.4]	
Black or African American	2073 (15.3) [15.0-15.5]	147 (12.8) [10.3-15.8]	136 (16.0) [13.4-19.0]	1790 (15.4) [15.0-15.8]	
White	9422 (70.8) [70.2-71.4]	782 (76.5) [73.3-79.3]	543 (71.0) [67.3-74.5]	8097 (70.2) [69.5-70.9]	
Hispanic ethnicity	3897 (22.2) [22.0-22.3]	188 (13.2) [11.3-15.3]	176 (17.7) [14.9-20.9]	3533 (23.4) [23.1-23.8]	<.001
US Census region					
Northeast	2037 (16.9) [16.8-17.0]	165 (16.6) [13.5-20.2]	126 (16.1) [13.2-19.5]	1746 (17.0) [16.5-17.4]	<.001
Midwest	2937 (21.6) [21.5-21.7]	266 (23.6) [20.0-27.7]	188 (23.0) [19.2-27.4]	2483 (21.3) [20.8-21.8]	
South	5136 (37.5) [37.4-37.7]	466 (44.5) [40.4-48.6]	308 (38.7) [34.6-42.9]	4362 (36.8) [36.3-37.3]	
West	3462 (24.0) [23.9-24.1]	177 (15.3) [12.5-18.6]	185 (22.2) [19.3-25.3]	3100 (24.9) [24.5-25.4]	
Age, y					
12-14	6972 (50.4) [50.3-50.6]	630 (57.3) [54.1-60.4]	352 (43.3) [39.9-46.9]	5990 (50.4) [50.1-50.8]	<.001
15-17	6600 (49.6) [49.4-49.7]	444 (42.7) [39.6-45.9]	455 (56.7) [53.1-60.1]	5701 (49.6) [49.2-49.9]	
No. of internalizing symptoms, mean (SD)^c^	1.80 (1.55)	1.95 (1.50)	1.79 (1.61)	1.78 (1.55)	.04
No. of non-ADHD externalizing symptoms, mean (SD)^d^	0.60 (0.76)	0.82 (0.85)	0.74 (0.85)	0.62 (0.73)	<.001
Lifetime nonmedical use of prescription stimulants	332 (2.5) [2.2-2.8]	91 (8.6) [6.9-10.6]	40 (4.7) [3.5-6.5]	201 (1.8) [1.6-2.0]	<.001
Past-year e-cigarette use	1184 (8.9) [8.3-9.5]	131 (12.7) [10.3-15.5]	106 (13.2) [10.5-16.5]	947 (8.2) [7.7-8.9]	<.001
Past-year cigarette smoking	1159 (8.6) [8.0-9.2]	162 (15.3) [13.1-17.7]	122 (14.6) [12.1-17.5]	875 (7.6) [7.0-8.2]	<.001
Past-year other tobacco use	1840 (11.4) [10.6-12.2]	201 (15.1) [12.9-17.7]	177 (17.2) [14.4-20.4]	1462 (10.6) [9.8-11.4]	<.001

Abbreviation: ADHD, attention-deficit/hyperactivity disorder.

^a^ Rates of missing items range from 0.01% (past-year other tobacco use) to 0.55% (ADHD diagnosis).

^b^ Continuous variables are compared using analysis of variance at the 95% level. Categorical variables are compared using Pearson χ^2^ test at the 95% level.

^c^ Ranges from 0 to 4.

^d^ Ranges from 0 to 3.

As shown in Table 2, the prevalence and incidence of all 4 nicotine and tobacco outcomes were highest among US youths who reported more ADHD symptoms. Across all 3 ADHD diagnosis and pharmacotherapy groups, most youths with highly symptomatic (3 or 4 symptoms) ADHD (range: 52.0% [95% CI, 49.9%-54.0%] to 58.6% [95% CI, 53.0%-64.0%]) reported incident e-cigarette use in the subsequent 9-year period. This compared with approximately one-third of youths with no symptoms in each of the 3 groups (range 31.4% [95% CI, 29.3%-33.5%] to 35.6% [95% CI, 27.3%-44.8%]). The prevalence and incidence of e-cigarette use, cigarette smoking, other tobacco use, or dual use among US youths with 1 or 2 ADHD symptoms consistently fell between the subgroup without symptoms and the subgroup with 3 or 4 ADHD symptoms for all nicotine and tobacco outcomes over a 9-year period. For instance, one-half of youths with highly symptomatic ADHD receiving pharmacotherapy reported incident dual use (50.8%; 95% CI, 45.3%-56.3%) in the subsequent 9-year period compared with a lower incidence of dual use among youths with an ADHD diagnosis taking pharmacotherapy with 1 or 2 ADHD symptoms (40.8%; 95% CI, 34.3%-47.8%) or no ADHD symptoms (24.2%; 95% CI, 18.6%-30.9%).

**Table 2.** ADHD Diagnosis, Number of Symptoms, Use of Pharmacotherapy, and Later Nicotine and Tobacco Use in US Youths^a^

Subgroup by symptoms at wave 1	Weighted % (95% CI)							
	E-cigarette use		Cigarette smoking		Other tobacco use		Dual use	
	Wave 1 prevalence (n = 13 336)	Waves 2-7 incidence (n = 12 091)^b^	Wave 1 prevalence (n = 13 336)	Waves 2-7 incidence (n = 11 722)^b^	Wave 1 prevalence (n = 13 336)	Waves 2-7 incidence (n = 11 700)^b^	Wave 1 prevalence (n = 13 336)	Waves 2-7 incidence (n = 12 417)^b^
**No ADHD diagnosis**								
No symptoms (n = 3123)	4.7 (4.0-5.5)	31.4 (29.3-33.5)	4.9 (4.1-5.8)	18.8 (17.3-20.3)	6.4 (5.5-7.5)	24.1 (22.2-26.1)	3.5 (2.9-4.3)	23.5 (21.8-25.3)
1 or 2 Symptoms (n = 4582)	7.1 (6.3-7.9)	41.6 (40.1-43.1)	6.1 (5.2-7.1)	23.5 (22.0-25.0)	9.1 (8.1-10.2)	31.7 (30.1-33.3)	5.0 (4.3-5.7)	31.0 (29.6-32.5)
3-4 Symptoms (n = 3970)	12.3 (11.3-13.4)	52.0 (49.9-54.0)	11.3 (10.3-12.4)	31.6 (29.6-33.7)	15.4 (10.5-22.3)	39.8 (37.8-41.8)	8.8 (7.9-9.8)	42.3 (40.2-44.5)
**ADHD diagnosis and no ADHD pharmacotherapy**								
No symptoms (n = 184)	9.8 (5.7-16.6)	35.6 (27.3-44.8)	11.9 (7.6-18.1)	27.6 (20.4-36.2)	15.5 (10.5-22.3)	33.0 (24.9-42.2)	8.5 (4.7-14.9)	29.6 (21.9-38.7)
1 or 2 Symptoms (n = 270)	11.8 (7.6-17.8)	42.3 (36.3-48.4)	12.6 (8.7-17.7)	31.1 (25.0-37.9)	17.0 (12.8-22.3)	38.5 (32.1-45.3)	9.1 (6.0-13.8)	36.7 (31.0-42.8)
3 or 4 Symptoms (n = 351)	16.0 (12.3-20.7)	56.8 (50.0-63.3)	17.6 (13.9-22.1)	35.7 (29.3-42.8)	18.3 (14.3-23.1)	48.7 (42.7-54.7)	13.0 (9.7-17.1)	49.5 (42.3-56.8)
**ADHD diagnosis and ADHD pharmacotherapy**								
No symptoms (n = 220)	7.6 (4.4-12.7)	31.8 (25.7-38.7)	8.3 (5.0-13.4)	21.8 (16.7-27.9)	9.5 (5.8-15.0)	30.2 (24.3-36.9)	6.2 (3.6-10.5)	24.2 (18.6-30.9)
1 or 2 Symptoms (n = 334)	11.9 (8.4-16.7)	49.5 (43.5-55.5)	12.7 (8.9-17.8)	35.1 (28.2-42.6)	12.1 (8.6-16.7)	40.8 (33.5-48.6)	9.4 (6.5-13.6)	40.8 (34.3-47.8)
3 or 4 Symptoms (n = 519)	15.1 (11.9-19.0)	58.6 (53.0-64.0)	19.8 (16.7-23.3)	39.3 (34.5-44.3)	19.4 (16.1-23.2)	46.6 (40.4-52.9)	12.3 (9.5-15.9)	50.8 (45.3-56.3)

Abbreviation: ADHD, attention-deficit/hyperactivity disorder.

^a^ Includes 13 572 participants. Wave 1 measured past 12-month prevalence during 2013 to 2014; waves 2 to 7 follow-up occurred from 2015 to 2023.

^b^ Incidence estimates exclude individuals who reported the particular outcome at wave 1 to establish new onset.

As shown in Table 3, the resulting sample sizes of the applicable subpopulations were 11 722 for cigarettes, 12 091 for e-cigarette, 11 700 for other tobacco products, and 12 417 for dual use. Discrete time survival models indicated that the adjusted odds of incident e-cigarette use, cigarette smoking, other tobacco use, and dual use did not significantly differ between youths with asymptomatic ADHD (whether they were receiving pharmacotherapy for ADHD or not) compared with population controls. In contrast, all 3 subgroups of youths with 3 or more ADHD symptoms had significantly greater adjusted odds of most incident outcomes compared with population controls. For instance, youths with ADHD receiving pharmacotherapy and reporting 3 or more past-year ADHD symptoms at wave 1 had greater odds of incident e-cigarette use (adjusted odds ratio [AOR], 1.60; 95% CI, 1.34-2.04), cigarette smoking (AOR, 1.52; 95% CI, 1.22-1.89), other tobacco use (AOR, 1.61; 95% CI, 1.27-2.02), and dual use (AOR, 1.72; 95% CI, 1.38-2.14) than population controls reporting no symptoms. Consistent with the bivariate results, the adjusted odds for US youths with 1 or 2 ADHD symptoms consistently fell between the subgroups without symptoms and 3 or more ADHD symptoms for all the nicotine and tobacco outcomes consistent with a dose-response association.

**Table 3.** ADHD Diagnosis, Use of Pharmacotherapy, and Number of Symptoms at Wave 1 and Odds of Later Nicotine and Tobacco Use at Waves 2 to 7 in US Youths

Subgroup by symptoms at wave 1	Incidence, AOR (95% CI)^a^			
	E-cigarette use (n = 12 091)	Cigarette smoking (n = 11 722)	Other tobacco use (n = 11 700)	Dual use (n = 12 417)
**No ADHD diagnosis**				
No symptoms	1 [Reference]	1 [Reference]	1 [Reference]	1 [Reference]
1-2 Symptoms	1.15 (1.05-1.27)^b^	1.03 (0.90-1.19)	1.16 (1.03-1.32)^b^	1.08 (0.97-1.19)
3-4 Symptoms	1.36 (1.20-1.54)^b^^,^^c^	1.21 (1.04-1.41)^b^	1.36 (1.17-1.59)^b^	1.32 (1.15-1.52)^b^^,^^c^
**ADHD diagnosis and no ADHD pharmacotherapy**				
No symptoms	1.12 (0.78-1.60)	1.39 (0.92-2.12)	1.17 (0.81-1.70)	1.14 (0.76-1.70)
1-2 Symptoms	1.13 (0.88-1.44)	1.35 (0.97-1.87)	1.35 (1.00-1.82)^b^	1.23 (0.94-1.61)
3-4 Symptoms	1.53 (1.18-1.99)^b^^,^^c^	1.32 (0.94-1.86)	1.68 (1.30-2.18)^b^	1.53 (1.17-2.01)^b^^,^^c^
**ADHD diagnosis and ADHD pharmacotherapy**				
No symptoms	0.98 (0.74-1.30)	1.01 (0.71-1.46)	1.25 (0.88-1.77)	0.92 (0.65-1.30)
1-2 Symptoms	1.42 (1.11-1.81)^b^^,^^c^	1.43 (1.05-1.95)^b^	1.45 (1.09-1.92)^b^	1.40 (1.05-1.86)^b^^,^^c^
3-4 Symptoms	1.60 (1.34-2.04)^b^^,^^c^^,^^d^	1.52 (1.22-1.89)^b^	1.61 (1.27-2.02)^b^	1.72 (1.38-2.14)^b^^,^^c^

Abbreviations: ADHD, attention-deficit/hyperactivity disorder; AOR, adjusted odds ratio.

^a^ Incidence estimates exclude individuals who reported the specified outcome at wave 1 (2013-2014) to establish new onset during the follow-up period of waves 2 to 7 (2015-2023). All models control for the age, sex, race, Hispanic ethnicity, region, other externalizing symptoms, internalizing symptoms, and nonmedical use of prescription stimulants.

^b^ Significantly different from the subgroup with no ADHD diagnosis and no ADHD symptoms.

^c^ Significantly different from the subgroup with ADHD diagnosis, ADHD pharmacotherapy, and no ADHD symptoms.

^d^ Significantly different from the subgroup with ADHD diagnosis, no ADHD pharmacotherapy, and no ADHD symptoms.

**Figure 1.**
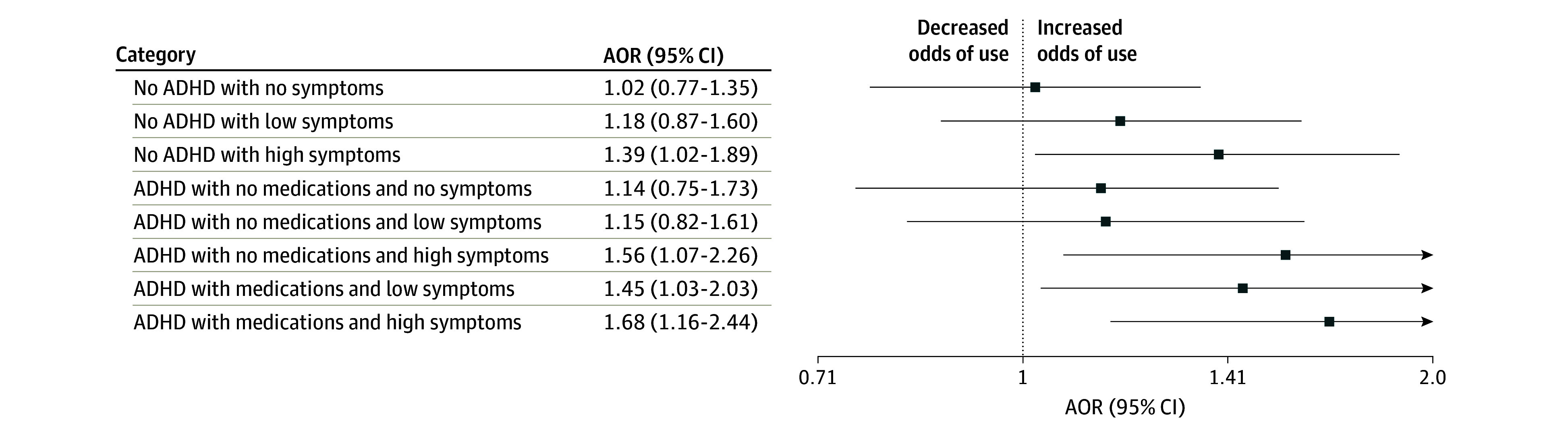
Association of E-Cigarette Use With Attention-Deficit/Hyperactivity Disorder (ADHD) Diagnosis, Number of Symptoms, and Use of Pharmacotherapy in US Youths (2015-2023) Low symptoms are defined as 1 or 2 symptoms; high, 3 or 4 symptoms. The reference was the subgroup with ADHD diagnosis with ADHD medications and no ADHD symptoms. AOR indicates adjusted odds ratio.

**Figure 2.**
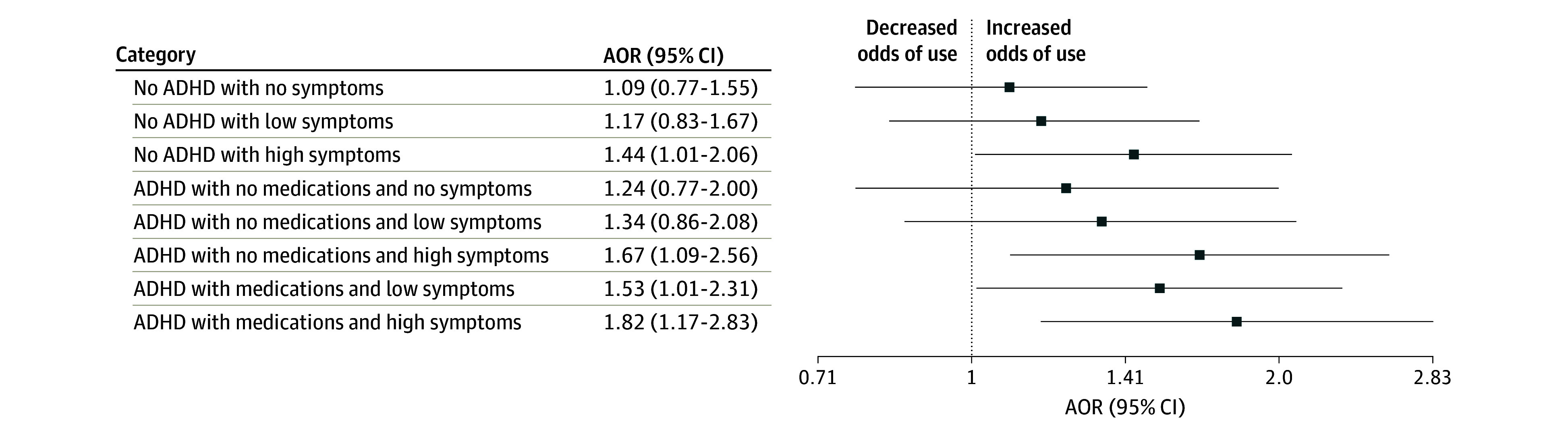
Association of Dual E-Cigarette and Tobacco Use With Attention-Deficit/Hyperactivity Disorder (ADHD) Diagnosis, Number of Symptoms, and Use of Pharmacotherapy in US Youths (2015-2023) Low symptoms are defined as 1 or 2 symptoms; high, 3 or 4 symptoms. The reference was the subgroup with ADHD diagnosis with ADHD medications and no ADHD symptoms. AOR indicates adjusted odds ratio.

Youths with no ADHD diagnosis and 3 or more ADHD symptoms had greater odds of incident e-cigarette use (AOR, 1.36; 95% CI, 1.20-1.54), cigarette smoking (AOR, 1.21; 95% CI, 1.04-1.41), other tobacco use (AOR, 1.36; 95% CI, 1.17-1.59), and dual use (AOR, 1.32; 95% CI, 1.15-1.52) compared with population controls (Table 3). A sensitivity analysis was conducted by excluding youths who reported any nicotine or tobacco use at wave 1 and the substantive results in Table 3 did not change. Youths with ADHD experiencing 3 or more ADHD symptoms and receiving pharmacotherapy were significantly more likely to initiate e-cigarette (AOR, 1.68; 95% CI, 1.16-2.44) and dual use (AOR, 1.82; 95% CI, 1.17-2.83) than youths with asymptomatic ADHD receiving pharmacotherapy (Figure 1 and Figure 2). In addition, youths with no ADHD diagnosis and 3 or more ADHD symptoms had significantly greater adjusted odds of incident e-cigarette use (AOR, 1.39; 95% CI, 1.02-1.89) and incident dual use (AOR, 1.44; 95% CI, 1.01-2.06) compared with youths with asymptomatic ADHD receiving pharmacotherapy (Figures 1 and 2).

## Discussion

The present cohort study found that US youths with asymptomatic ADHD—with or without ADHD pharmacotherapy—did not differ from population controls in their odds of initiating e-cigarette use, cigarette smoking, other tobacco use, or dual use over the subsequent 9 years. In contrast, US youths who reported higher levels of ADHD symptoms—regardless of lifetime ADHD diagnosis or pharmacotherapy—had significantly greater odds of initiating e-cigarette use, cigarette smoking, other tobacco use, or dual use in the subsequent 9 years.

Like prior studies of ADHD symptoms and cigarette smoking,^7,8^ the odds of initiating e-cigarette use, cigarette smoking, other tobacco use, or dual use, increased with ADHD symptom severity in a dose-response association, with the subgroups without symptoms demonstrating the lowest risk, those with lower ADHD symptom severity (1-2 symptoms) demonstrating intermediate risk, and those with higher ADHD symptom severity (3-4 symptoms) demonstrating the highest risk. Potential reasons for this increased risk are that youths with higher symptom severity may use nicotine to self-treat their ADHD symptoms. Nicotine has been shown to enhance cognition in general and reduce ADHD symptoms specifically.^36^ Moreover, it has been speculated that nicotine may increase the release of several neurotransmitters that via nicotinic receptors increase catechol, indolamines, β-endorphin, and γ-aminobutyric acid levels, potentially mediating the neurobiological deficits inherent in ADHD.^37,38^

The findings in the present study add to existing literature in compelling ways and have important clinical and policy implications. First, the results highlight the significant longitudinal association between ADHD symptom severity and future nicotine and tobacco use. Most US youths who reported 3 or more ADHD symptoms in the present study at wave 1 initiated e-cigarette use in the subsequent 9-year period, which was not observed among those with 0 to 2 symptoms. Second, the finding that US youths with asymptomatic ADHD—with or without pharmacotherapy—did not differ from their population control peers without ADHD diagnoses in their odds of initiating e-cigarette use, cigarette smoking, other tobacco use, or dual use over the subsequent 9-year period highlights the importance of monitoring and management of ADHD symptoms and the potential protective effect of treatment against nicotine and tobacco use.

A major clinical implication of these findings is that health care professionals need to closely monitor inattentive and hyperactive and/or impulsive symptoms and work to reduce them to the lowest levels. While more research is needed to determine the degree to which ADHD symptom reduction lowers risk behaviors, there is a growing body of literature showing that greater symptom reduction through treatment results in improved functional outcomes,^39^ while persistence of ADHD symptoms can result in adverse outcomes.^40^ The present findings support the assertion from prior clinical studies that alleviating core ADHD symptoms may lead to a reduction in risky behaviors among US youths with ADHD.^22,41^

Given the increased risk of nicotine and tobacco use in those with ADHD,^1,2,3^ the null findings between youths with asymptomatic ADHD and later nicotine and tobacco initiation could be interpreted as ADHD treatment being protective against subsequent nicotine and tobacco use. Indeed, those with no ADHD diagnosis and higher ADHD symptom severity were at greater risk for nicotine and tobacco onset than youths with asymptomatic ADHD. Taken together, these findings underscore the risk associated with higher levels of inattentive and hyperactive and/or impulsive symptoms and the need to identify such youths as early as possible so that they may access services to reduce symptoms. The group of adolescents with inattentive and hyperactive and/or impulsive symptoms and no ADHD diagnosis represents a high-risk subpopulation often missed in clinical research. Pediatric health care professionals should be knowledgeable about ADHD symptoms, screening and assessment tools, and treatment options, including stimulant and nonstimulant medications and psychosocial interventions that reduce inattentive and hyperactive and/or impulsive symptoms.^42,43^ In addition, parents and caregivers should be educated on ADHD symptoms, screening and assessment tools, and treatment options.

### Strengths and Limitations

There are several notable strengths of the present study, including the use of a large nationally representative longitudinal sample of US youths whose ADHD diagnosis, symptoms, and pharmacotherapy were assessed using the same methodology over time. The study featured population controls without ADHD and assessed multiple forms of nicotine and tobacco use to better understand the current landscape of substance use among US youths, including newer e-products.

There were also some limitations that must be weighed when considering the implications of the findings. The list of ADHD symptoms that was assessed included several items from validated and reliable screeners recommended in the PhenX Toolkit^28^ but did not include an exhaustive list of all ADHD symptoms. Screening instruments based on self-reports are designed to quickly identify individuals who have a potential diagnosis and may need more comprehensive follow-up clinical assessment. Prior work has shown screening tools such as the GAIN Short Screener should not be used by clinicians to establish a specific diagnosis on its own due to low specificity and the risk of misclassifying individuals.^26,44^ The sample included individuals 12 years and older, and some had already initiated nicotine or tobacco use at wave 1. While examining incident use (ie, rather than overall prevalence) partially addressed this limitation, future longitudinal research should begin assessments for ADHD symptoms and nicotine and tobacco use at earlier ages. Finally, the COVID-19 pandemic had a major impact on ADHD diagnosis, treatment, and substance use among US youths.^14,45,46,47,48,49^ In recent years, there have been shortages of some ADHD medications that, along with disruptions caused by the COVID-19 pandemic, have impacted ADHD treatment for many US youths (S. He, S.E.M., R Conti, A. Volerman, K. P. Chua; unpublished data; completed September 23, 2024).^49^ While we examined nicotine and tobacco initiation after onset of the COVID-19 pandemic, future research is warranted to examine potential changes in ADHD diagnosis, treatment, and the associations between ADHD and substance use onset, specifically during and following the COVID-19 pandemic.

## Conclusions

In this cohort study of US youths, the findings support prior research and provide more current information about the associations between ADHD symptoms and newer forms of nicotine and tobacco use. Findings highlight the importance of early diagnosis and effective treatment of ADHD—with or without pharmacotherapy—to alleviate ADHD symptoms and thus reduce the risk of later nicotine and tobacco use among US youths. Future research should consider similar methods to examine associations between ADHD treatment, symptoms, and other substance use and adverse health outcomes to better understand whether a threshold of inattentive and hyperactive and/or impulsive symptoms exists that can be clinically monitored to improve health outcomes. This line of research is needed to guide clinicians in treating ADHD and to prevent the onset and escalation of nicotine and tobacco use among this high-risk population.

## Notes

Supplement.
Data Sharing Statement
