# Supplementary material for: Attention-Deficit/Hyperactivity Disorder Symptoms and Later E-Cigarette and Tobacco Use in US Youths
Source: JAMA Netw Open. 2025 Feb 11;8(2):e2458834. doi: 10.1001/jamanetworkopen.2024.58834 (PMC11815522; doi:10.1001/jamanetworkopen.2024.58834)
Supplement: Supplement. — Data Sharing Statement [file jamanetwopen-e2458834-s001.pdf]

Data Sharing Statement

McCabe. Attention-Deficit/Hyperactivity Disorder Symptoms and Later E-Cigarette and Tobacco Use in US Youths. *JAMA Netw Open*. Published February 11, 2025. doi:10.1001/jamanetworkopen.2024.58834

Data
Data available: Yes
Data types: Other (please specify)
Additional Information: The data used in this study can be accessed through the National Addiction and HIV Data Archive Program.
How to access data: The data used in this study can be accessed through the National Addiction and HIV Data Archive Program at https://www.icpsr.umich.edu/web/pages/NAHDAP/index.html.
When available: With publication

Supporting Documents
Document types: None

Additional Information
Who can access the data: Anyone can request the restricted data used in this study via the National Addiction and HIV Data Archive Program at https://www.icpsr.umich.edu/web/pages/NAHDAP/index.html.
Types of analyses: Any use.
Mechanisms of data availability: With a data use agreement.
